# Hierarchical Boosting Dual-Stage Feature Reduction Ensemble Model for Parkinson’s Disease Speech Data

**DOI:** 10.3390/diagnostics11122312

**Published:** 2021-12-09

**Authors:** Mingyao Yang, Jie Ma, Pin Wang, Zhiyong Huang, Yongming Li, He Liu, Zeeshan Hameed

**Affiliations:** 1 College of Microelectronics and Communication Engineering, Chongqing University, Chongqing 400000, China; zgyangmy@gmail.com (M.Y.); 202012021059t@cqu.edu.cn (J.M.); wangpin@cqu.edu.cn (P.W.); zeeshanhameed.zh@gmail.com (Z.H.); 2 Chongqing Academy of Educational Sciences, Chongqing 400000, China; bjxiexintong@163.com

**Keywords:** Parkinson’s disease, hierarchy space instance learning mechanism, dual-stage feature reduction pair, ensemble learning

## Abstract

As a neurodegenerative disease, Parkinson’s disease (PD) is hard to identify at the early stage, while using speech data to build a machine learning diagnosis model has proved effective in its early diagnosis. However, speech data show high degrees of redundancy, repetition, and unnecessary noise, which influence the accuracy of diagnosis results. Although feature reduction (FR) could alleviate this issue, the traditional FR is one-sided (traditional feature extraction could construct high-quality features without feature preference, while traditional feature selection could achieve feature preference but could not construct high-quality features). To address this issue, the Hierarchical Boosting Dual-Stage Feature Reduction Ensemble Model (HBD-SFREM) is proposed in this paper. The major contributions of HBD-SFREM are as follows: (1) The instance space of the deep hierarchy is built by an iterative deep extraction mechanism. (2) The manifold features extraction method embeds the nearest neighbor feature preference method to form the dual-stage feature reduction pair. (3) The dual-stage feature reduction pair is iteratively performed by the AdaBoost mechanism to obtain instances features with higher quality, thus achieving a substantial improvement in model recognition accuracy. (4) The deep hierarchy instance space is integrated into the original instance space to improve the generalization of the algorithm. Three PD speech datasets and a self-collected dataset are used to test HBD-SFREM in this paper. Compared with other FR algorithms and deep learning algorithms, the accuracy of HBD-SFREM in PD speech recognition is improved significantly and would not be affected by a small sample dataset. Thus, HBD-SFREM could give a reference for other related studies.

## 1. Introduction

Parkinson’s disease (PD) is a neurodegenerative disease with the characteristics of motor stiffness, movement retardation, tremor, and some non-motor symptoms (NMS, like bass disorder, sleep disorder, depression, constipation, pain, and dysarthria). Numerous studies have shown that PD patients will have NMS as the disease develops that seriously affects the quality of life [1].

NMS can be detected at the early stage of the disease, which allows a sound treatment plan to be designed. Dysarthria is the primary NMS and plays a guiding role in the study of PD pathogenesis. In addition, the advantages of speech data collection have made speech analysis gradually become the main analysis method for PD recognition as well as a key research area for early PD recognition [2].

However, speech data exhibit a high rate of redundancy and repetition and contain much unnecessary noise. Feature reduction (FR) could help alleviate this issue. Currently, this topic has attracted extensive attention from researchers and has great research significance [3]. Early FR research on PD speech recognition primarily focused on feature selection, which could be simply considered as the way of selecting the optimal feature subset from the original feature space. Some feature selection algorithms include Relief [4,5,6], (mRMR) [3], SBS [7], PSO [8], SFS [9], LASSO [2,4], Pvalue [10]. Erika R et al. selected the optimal subset of features from the original features, then using the Pvalue algorithm [10]. Sakar and Kursun [11] proposed a new feature selection algorithm based on mutual information, and the model is trained using support vector machines (SVM), achieving an accuracy of 92.75%. Musa Peker [12] used mRMR to identify valid features and then submitted the obtained features into a complex-valued artificial neural network. Benba et al. [13] selected features based on pathology thresholds through a multi-dimensional voice detection procedure (MDPV) and then submitted the obtained features to K-nearest neighbors (KNN) and SVM, achieving an accuracy of 95%. Shirvan RA et al. [14] used genetic algorithms and KNN to determine the optimal features that affected the result of recognition.

Feature extraction is another type of FR algorithm, the idea is to map the high-dimensional features to the low-dimensional space and keep all the information of the original instance as much as possible [15]. Linear approaches were primarily used before, in which PCA [16,17], and LDA [18,19,20,21] were representative methods. Chen et al. [21] developed a PD detection system that used PCA to extract features and trained the model with a fuzzy KNN classifier, which achieved an accuracy of 96.7%. Hariharan M et al. extracted features of PD using PCA and LDA and obtained a high accuracy rate [10]. Linear feature extraction methods generally assume the data in a high-dimensional linear space, which is the opposite of the non-linear characteristics of PD speech datasets in the real world [22,23,24]. Thus, linear feature extraction could not be applied well to non-linear data spaces because it limits the accuracy of PD recognition [25]. Currently, non-linear feature extraction has been developed and applied to PD recognition [19,26,27]. Kernel mapping and deep neural network mapping are two representative types of non-linear feature extraction methods. Yang achieved good results by feature extraction of PD speech data through SFS and PCA with kernel [19]. Derya A proposed the Genetic Algorithm-Wavelet Kernel-Extreme Learning Machine (GA-WK-ELM), and the wavelet kernels were used to map non-linear features from PD speech data [25]. Grover used deep neural networks to process Parkinson’s disease speech data features and predict the severity of PD [26]. Camilo considered multimodal information, including not only speech data of PD patients, but also writing, handwriting data and gait, and posture data and trained the model for recognition according to deep learning methods [27].

Manifold learning is another type of feature extraction method that could be applied to small sample datasets. Locally preserved projection (LPP) is a representative algorithm for manifold learning, which preserves the structure of the nearest neighbor between data samples after feature extraction, while minimizing the dimensions of the features [28]. However, since LPP is the nearest neighbor retention algorithm, most of the improved algorithms based on LPP only focus on the differences between classes and do not consider the large differences within classes [29,30,31]. Liu et al. considered both interclass data aliasing and intraclass data aliasing, which effectively solve these problems [16].

In recent studies, some scholars have attempted to integrate the advantages of feature selection and feature extraction to create hybrid feature processing methods. M. Hariharan et al. [9] proposed a hybrid system using SFS and PCA to process the data feature characteristics and feed the processed bibliography into a least square support vector machine classifier to learn the prediction model. H. Almayyan et al. [32] proposed a hybrid recognition system that uses PCA and Relief for feature processing and SVM combined with recursive feature elimination (SVMRFE) as a classifier to train the model. In addition, the study still used the SMOTE technique in order to equalize and diversify the dataset.

Based on the above analysis, we know that the FR method can solve the problems of high redundancy, high repetition, and noise of speech data. However, traditional feature extraction could construct high-quality features but could not achieve feature preference, while traditional feature selection could achieve feature preference but could not construct high-quality features. The two types of FR methods are different in principle but can be complementary to each other. Thus, it is necessary to propose a feature reduction method that could simultaneously achieve feature preference and high-quality feature construction. Although some related studies have made some progress in this field [21,32], critical problems also remain to be solved: (1) the integration of feature extraction and feature selection always occurs once, then the absence of multiple iterations to find the optimal fusion make it impossible to obtain higher quality merged features; (2) existing methods only consider information on the characteristics of the sample in the original space, and ignoring structural information on the characteristics of the deeper instance. In order to address these issues, the Hierarchical Boosting Dual-Stage Feature Reduction Ensemble Model for Parkinson’s disease speech data (HBD-SFREM) is proposed in this study. The major contributions and innovations of this model are listed below.

1. The instance space of the hierarchy is built by an iterative deep extraction mechanism.
2. The manifold feature extraction method embeds the nearest neighbor feature preference method to form a dual-stage feature reduction pair module.
3. The dual-stage feature reduction pair (D-Spair) module is iteratively performed by the AdaBoost mechanism to obtain higher quality features, thus achieving a substantial improvement in model diagnosis accuracy.
4. The deep hierarchy instance space is integrated into the original instance space to enhance the generalization ability of the model.

The writing structure of this paper is given here. Section 2 introduces the principles related to the proposed model; Section 3 describes the experiments designed in this paper as well as the presentation and analysis of the results; Section 4 analyzes the limitations and contributions of this study.

## 2. Materials and Methods

### 2.1. Symbol Description

In order to facilitate the presentation of the HBD-SFREM, some symbols need to be defined first. The datasets used in this study are numerical matrices and described as $X={\left[x_{1},x_{2},\ldots ,x_{N}\right]}^{T}={\left[X^{1},X^{2},\ldots ,X^{C}\right]}^{T}\in R^{N\times D}$, where $N=N_{1}+N_{2}+\cdots +N_{C}$. By default, each row represents an instance, N indicates the number of instances in X. D denotes the dimension of X. C is the category of datasets, the label of instances is expressed as $y={\left[y_{1},y_{2},\cdots ,y_{N}\right]}^{T}\in R^{N}$. The number of instances in each hierarchy is determined by the number of instances in the upper hierarchy and P, where P is the proportion of instances retained when IDEM is performed. The mapping matrix generated by the D-Spair maps $R^{D}$ to $R^{d}$, where $R^{D}$ represents the high-dimensional dataset, and $R^{d}$ represents the low-dimensional dataset, (d<D).

### 2.2. The Proposed Algorithm

#### 2.2.1. Construction of the Different Hierarchy Instance Space

In this part, the layers H of hierarchical instance spaces and the numbers of independent instance subspace n are used. One of the primary innovations in this paper is that deep hierarchy instance space is constructed based on IDEM. The relationship between the different hierarchies of instance spaces is analyzed by learning instances of different hierarchy spaces, and the generalization ability of the final model will also be improved.

In the IDEM mechanism, $\pi_{j}$ is used to define the clusters and the clustered partition of data points is denoted by ${\left\{\pi_{j}\right\}}_{j=1}^{k}$, while the radial basis function ϕ is used to map the data to high-dimension space, thus the objective function is defined as:

(1) $$D({\left\{\pi_{j}\right\}}_{j=1}^{k})=\sum_{j=1}^{k}\sum_{a\in \pi_{j}}w(a)||\phi (x_{i})-m_{j}{\left|\right|}^{2},m_{j}=\frac{\sum {}_{b_{i}\in \pi_{c}}w(b)\phi (x_{i})}{\sum {}_{b_{i}\in \pi_{c}}w(b)|\pi_{j}|}$$

where $m_{j}$ is the center of each cluster, where $x_{i}$ is instances of $X_{train}$.

Assume that each cluster has the same weight, the Euclidean distance of each sample ϕ(s) to the cluster center $m_{j}$ is denoted as:

(2) $${\left\|\phi \left(x_{i}\right)-\frac{\sum {}_{a_{i}\in \pi_{c}}\phi (x_{i})}{\left|\pi_{j}\right|}\right\|}^{2}=\phi \left(x_{i}\right)\cdot \phi \left(x_{i}\right)-\frac{2\sum_{x_{j}\in \pi_{c}}\phi \left(x_{i}\right)\cdot \phi \left(x_{j}\right)}{\left|\pi_{c}\right|}+\frac{\sum_{x_{j},x_{l\in \pi_{c}}}\phi \left(x_{j}\right)\cdot \phi \left(x_{l}\right)}{\left|\pi_{c}\right|},\;\phi \left(x_{i}\right)\cdot \phi \left(x_{j}\right)=\phi \left(x_{i}\cdot x_{j}\right)$$

**Figure 1:**
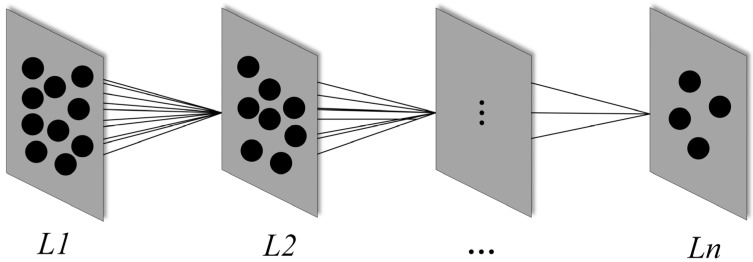
Flow chart of IDEM.

Figure 1 describes the detailed process of the IDEM. The IDEM is based on the means clustering method with radial basis kernel [33,34,35]. The original dataset is defined as the first hierarchy instance, and the IDEM mechanism is used to cluster this hierarchy instance to generate the second hierarchy instance. Then, the second hierarchy instance is clustered to generate the third hierarchy instance, until the H-th hierarchy instances are generated, where $H\in n^{+}$ ($n^{+}$ represents the set of positive integers). The number of newly generated instances is P% from the upper hierarchy instances.

#### 2.2.2. Boosting Dual-Stage Feature Reduction Pair Ensemble Module

The typical characteristics of PD speech datasets are a small sample, having high repetition, high redundancy, and a certain amount of noise. According to the characteristics above, the boosting dual-stage feature reduction pair ensemble module (BD-SFREM) is designed to address this issue, which includes the dual-stage feature reduction pair (D-Spair) module and boosting ensemble module.

1. D-Spair module;

Suppose the number of instances of $c_{th}$ is $N_{c}$, then the total number of the instance $k_{n}=\sum_{c=1}^{C}k_{nc}$. In the first step, D-Spair makes instances belonging to the same category closer together after mapping, that is, the within-class variance matrix of similar samples is reduced, the specific mathematical formula is expressed as follows:

(3) $$\begin{array}{l} \underset{M}{min}{\sum_{c=1}^{C}\left\|M^{T}x^{\left(c\right)}-M^{T}\bar{x_{w}^{\left(c\right)}}\right\||}_{x^{\left(c\right)}\in x_{wc}}\\ =\underset{M}{min}M^{T}S_{SC}M \end{array},$$

where $S_{SC}=\sum_{c=1}^{C}(x^{\left(c\right)}-\bar{x_{w}^{\left(c\right)}}){\left(x^{\left(c\right)}-\bar{x_{w}^{\left(c\right)}}\right)}^{T}$ stands for the variance matrix of the intraclass. $\bar{x^{\left(c\right)}}=\frac{1}{k_{nc}}\sum_{i=1}^{k_{nc}}x_{i}^{\left(c\right)}$ denotes the center of c-th class, and $x_{wc}$ the samples belonging to the same class.

Similarly, instances with different class labels are mapped as far apart as possible, that is the variance matrix between different classes should be increased as much as possible, and the specific mathematical formula is expressed as follows:

(4) $$\begin{array}{l} \underset{M}{max}{\sum_{c=1}^{C}\left\|M^{T}\bar{x_{b}^{\left(c\right)}}-M^{T}\bar{x_{b}}\right\||}_{x_{b}^{\left(c\right)},x_{b}\in X_{DC}}\\ =\underset{M}{max}M^{T}S_{DC}M \end{array},$$

where $S_{DC}=\sum_{c=1}^{C}(\bar{x_{b}^{\left(c\right)}}-\bar{x_{b}}){\left(\bar{x_{b}^{\left(c\right)}}-\bar{x_{b}}\right)}^{T}$ represents the scatter matrix between different classes. $\bar{x_{b}}=\frac{1}{k_{n}}\sum_{i=1}^{k_{n}}x_{i}$ stands for the center of the local part, and $\bar{x_{b}^{\left(c\right)}}=\frac{1}{N_{k}}\sum_{i=1}^{N_{k}}x_{i}^{\left(c\right)}$ the number of the c-th class in the local part.

In addition, the nearest neighbor structure between samples is preserved during the mapping process (i.e., locality preservation), the specific mathematical formula could be described as follows:

(5) $$\begin{array}{l} \sum_{c=1}^{C}\sum_{i=1}^{N_{c}}\sum_{j=1}^{N}Z_{ij}^{c}{{\left\|M^{T}x_{i}^{\left(c\right)}-M^{T}x_{j}\right\|}^{2}|}_{x_{i}^{\left(c\right)},x_{j}\in X_{train}}\\ =M^{T}X_{train}AX_{train}^{T}M \end{array},$$

where A=U−Z represents a Laplacian matrix, $U_{ij}^{c}=\sum_{j}Z_{ij}^{c}$ a diagonal matrix and the elements are the result of summing the diagonal elements of Z, $Z=\{\begin{array}{lll} Z^{1} & & \\ & \ddots & \\ & & Z^{C} \end{array}\}$ stands for an affinity matrix, $U=\{\begin{array}{lll} U^{1} & & \\ & \ddots & \\ & & {\;U}^{C} \end{array}\}$.

Thus, the objective function of the feature extraction part of the D-Spair is designed to minimize the local variance matrix within the same category and maximize the variance matrix between different categories, while preserving the nearest neighbor structure of each instance. Based on the description of Equations (3)–(5), the mathematical expression of the feature extraction part is expressed as follows.

(6) $$\underset{M}{min}Tr(\frac{M^{T}S_{SC}M+M^{T}X_{train}AX_{train}^{T}M}{M^{T}S_{DC}M}),$$

Equation (6) could be transformed by the Lagrange multiplier method into Equation (7)

(7) $$L(M,\lambda )=M^{T}(S_{SC}+\lambda (\gamma X_{train}AX_{train}^{T}-\mu S_{DC}))M,$$

Take the derivative of M to obtain the optimal solution.

(8) $$\begin{array}{l} {\frac{\partial L(M,\lambda )}{\partial M}|}_{{}_{x_{i},x_{j}\in X_{train}}}=0\\ \Leftrightarrow {\left(\mu S_{DC}-\gamma X_{train}AX_{train}^{T}\right)}^{-1}S_{SC}M=\lambda M \end{array},$$

where λ and γ is the penalty factor. Equation (8) could be solved and the projection matrix M is obtained. The vector $M\in R^{D\times d}$ is the generalized eigenvector of ${\left(\mu S_{DC}-\gamma X_{train}AX_{train}^{T}\right)}^{-1}S_{SC}$ and λ is the first d largest eigenvalues. The vector $M_{k}=(m_{1},m_{2},\cdots ,m_{k})$ is composed of the first k eigenvectors of M.

Next, the vector $M_{k}$ is used to map $X_{train}$, resulting in high-quality feature extraction, the mapped data are named $X_{train}^{\prime }$. Define the sample set $X_{train}^{\prime }$ as S, divide $X_{train}^{\prime }$ into $S^{+}$ and $S^{-}$ according to the class label of instances. An instance $X_{i}$ is randomly selected from S without putting back ($X_{i}\in S$). According to the nearest neighbor criterion, an instance is also selected from $S^{+}$ and $S^{-}$ respectively, which are noted as $nearst_{i+}$, $nearst_{i-}$. Assume that $X_{i}$ has p features, i.e., each $X_{i}$ consists of p-dimensional vectors ($x_{i}^{1},x_{i}^{2},\cdots ,x_{i}^{p}$), where $x_{i}^{j}$ is the j−th feature of $X_{i}$.

Similarly, $W_{i}$ denotes the feature weight of $X_{i}$, which also consists of p-dimensional vectors $\left(w_{i}^{1},w_{i}^{2},\cdots ,w_{i}^{p}\right)$, $w_{i}^{j}$ denotes the feature weight of $x_{i}^{j}$. Same as $X_{i}$, $nearst_{i+}$ and $nearst_{i-}$ are also composed of p-dimensional vectors. Firstly, initialize the weights $W_{i}=(w_{i}^{1},w_{i}^{2},\cdots ,w_{i}^{p})=(0,0,\cdots ,0)$. Second, update $w_{i}^{j}$ according to the distances of $x_{i}^{j}$ from $nearst_{i+}^{j}$ and $nearst_{i-}^{j}$. The feature weights $W_{i}$ of a single instance are obtained by iterating p times. The feature weights W of all instances are obtained by iterating the above process m times. Finally, the higher quality features are selected with W that are useful to the training model. The related mathematical expressions are as follows:

(9) $$w_{i}^{j}=w_{i}^{j}+\left\|x_{i}^{j}-nearst_{i-}^{j}\right\|-\left\|x_{i}^{j}-nearst_{i+}^{j}\right\|,j=1,2,\cdots ,p,$$

(10) $$W=\frac{\sum_{i=1}^{m}W_{i}=\sum_{i=1}^{m}(w_{i}^{1},w_{i}^{2},\cdots ,w_{i}^{j},\cdots ,w_{i}^{p})\;}{m}$$

Then, these optimal features are used to train the classifier.

2. Boosting ensemble module;

In the boosting ensemble module, the AdaBoost mechanism is used to combine various D-Spair, thereby constructing the boosting ensemble module. Finally, the pseudocode of BD-SFREM is shown as follows.
**BD-SFREM****Input:** $X_{train}$: training dataset (an N×D matrix) and the corresponding labels $Y_{train}$ (an N×1 matrix) $X_{valid}$: valid dataset (an N×D matrix) and the corresponding labels $Y_{Valid}$ (an N×1 matrix) $X_{test}$: test data (an N×D matrix) and the corresponding labels $Y_{test}$ (an N×1 matrix) T: boosting module usage times Threshold: flag of boosting module end **Output:** Final Prediction $P_{K}^{final\_i}$ of the independent instance space
**Begin** 1: Given the data: $(x_{1},y_{1}),\ldots ,(x_{m},y_{m})\;,where\;x_{i}\in X_{train}\;,y_{i}\in Y_{train}=\left\{-1,+1\right\}$. 2: Initial weights of $X_{train}$:D1(i)=1m 3: **while** $e_{t}^{D-Spair}$ <= Threshold (where $e_{t}^{D-Spair}$ is error calculated from misclassified instances, t=1,2,⋯T) **do** 4:  Use the D-Spair module to obtain dual-stage features. 5:  Obtain weakclassifier, use dual-stage features: $h_{t}^{D-Spair}:X_{train}\to \left\{-1,+1\right\}with\;eror$. 6: Obtain misclassification instances rate: $e_{t}^{D-Spair}=P(h_{t}{}^{\;D-Spair}(x_{i})\neq y_{i})$ use $X_{valid}$, and obtain the misclassification instances. 7: Obtain weight of weak hypothesis $\alpha_{t}^{\;D-Spair}=\frac{1}{2}\log\frac{1-e_{t}^{\;D-Spair}}{e_{t}^{\;D-Spair}}$ 8: Add misclassified instances to $X_{train}$ form a new training set. 9: **End while** 10: **Obtain** the final prediction use $X_{test}$:   
$P_{K}^{final\_i}=sign(\sum_{t=1}^{T}\alpha_{t}^{\;D-Spair}h_{t}^{\;D-Spair}(x))$
**End**

#### 2.2.3. Hierarchical Space Instance Learning Mechanism

The implementation of the hierarchical space instance learning mechanism is based on the construction of the different hierarchy spaces and BD-SFREM. First, the IDEM mechanism is used to construct the deep hierarchy space. Then, the BD-SFREM is applied to different hierarchy spaces to perform the hierarchy space instance learning mechanism, and the results of the deep hierarchy spaces are integrated with the results of the original hierarchy spaces in order to improve the generalization ability of the model.

The pseudocode of the hierarchical space instance learning mechanism is shown as follows:
**Hierarchical space instance learning mechanism****Begin** 1: **For** d = 1:H **do** 2: $\left[\alpha ,\beta ]=size(X_{train}\right)$; //get the dimension information of $X_{train}$. 3: The number of output instance clusters: k=α×P. 4: Define the size of the cluster: $D^{\prime }\in R^{k\times D}$, where k stands for the number of centroid samples of cluster and D the dimensionality of the centroid samples.  $D\prime ({\left\{\pi_{j}\right\}}_{j=1}^{k})=\sum_{j=1}^{k}\sum_{a\in \pi_{j}}w(a)||\phi (x_{i})-m_{j}{\left|\right|}^{2}$
  
$\begin{array}{l} m_{j}=\frac{\sum {}_{b_{i}\in \pi_{c}}w(b)\phi (x_{i})}{\sum {}_{b_{i}\in \pi_{c}}w(b)|\pi_{j}|} \end{array}$
  
$A^{\left(k\right)}=\frac{1}{k}\sum_{j=1}^{k}a_{j}$ where A is the labels of the output samples after IDEM, and $a_{j}$ represents the labels that belong to the same category. 5: Obtain the H-th hierarchy: $X_{train}^{d}=[D^{\prime },A]$.  **End for** 6: Obtain the different hierarchy space construct by IDEM: $X_{train}^{H}=[X_{train}^{1},X_{train}^{2},\cdots ,X_{train}^{d}]$. 7: Apply BD-SFREM on $X_{train}^{H}$. 8: Obtain the $P_{K}^{final\_i}$ of the different hierarchy space. **End**

#### 2.2.4. Overall Description of the Proposed Model

**Figure 2:**
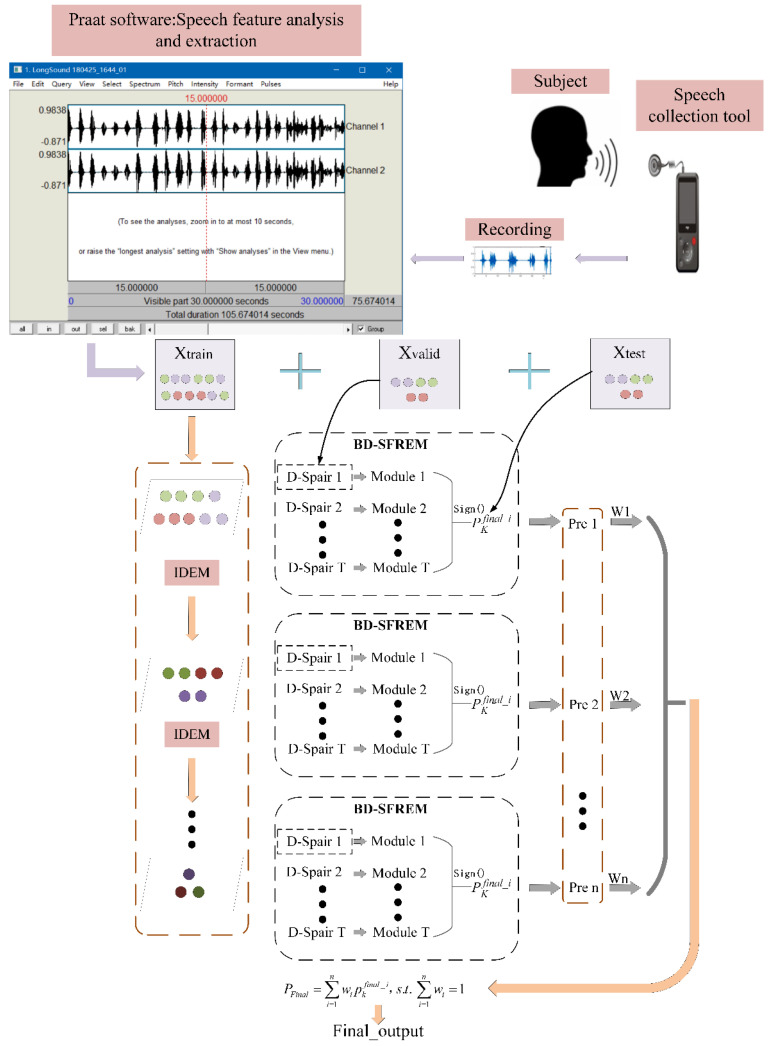
Graphical description of the proposed model.

The overall description of the proposed model (HBD-SFREM) is described in this part. First, the different hierarchy space is constructed by IDEM. Second, a method of boosting dual-stage feature reduction process (boosting dual-stage feature reduction pair ensemble module) is established based on the proposed objective function. Finally, the above methods are applied to different hierarchy spaces to perform hierarchy space instance learning, then the results of the deep hierarchy spaces are integrated with the results of the original hierarchy instance spaces in order to improve the generalization ability of the algorithm. Figure 2 depicts the algorithm of this paper.

## 3. Results

### 3.1. Datasets

Three representative PD speech datasets and a self-collected PD speech dataset were utilized to validate the innovation of the HBD-SFREM.

LSVT: The LSVT dataset was founded by Professor Athanasios Tsanas of the University of Oxford (tsanasthanasis@gmail.com). The role of this dataset was to assess effectiveness after rehabilitation treatment. In total, 14 subjects with PD (eight of them were male and six were female) participated in the entire data collection process. For more details, see [36].

PSDMTSR: The dataset consisted of a total sample of 40 subjects, in which 20 samples were from people with PD and 20 samples were from healthy people. For more details, see [37].

Parkinson: A total of 31 subjects’ speech data were collected in this dataset, 23 of whom were people with PD and eight of whom were healthy. For more details, see [38].

SelfData: The dataset was collected from a total of 31 subjects, 10 of whom suffered from PD and 21 of whom were healthy. Specifically, five of the 10 with PD were male and five were female; 12 of the 21 healthy subjects were male and nine were female. Thirteen voice segments (samples) were collected for each subject, and each voice segment consisted of 26 features. The SONY ICD-SX2000 recording tool was used for voice acquisition, and the recording tool was kept at a distance of 15 cm from the subject’s lips during the acquisition. Each subject was asked to read a specific piece of pronunciation material and the pronunciation made by each subject was recorded. The sampling was set to a frequency of 44.1 kHz and the resolution was set to 16 bits.

Three of the four datasets (LSVT, PSDMTSR, and Parkinson) are available to the public and can be downloaded from the UCI dataset repository created by the University of California, Irvine (www.archive.ics.uci.edu/ml/index.php (accessed on 24 November 2021)). The Chinese Army Medical University provided the SelfData dataset. Brief information about the datasets is shown in Table 1.

### 3.2. Experimental Environment

All experiments were conducted in MATLAB version 2017b, running on a PC with Windows 10, 64-bit and the CPU was intel(R) Core i5-2300 (2.80 GHz) as well as 8 GB of RAM. Praat is a computer speech processing software, which is used to analyze the speech features and extract speech features in this paper. The basic classifiers used in this study was the SVM. For optimal performance of the D-Spair, the affinity matrix Z was constructed using adjustable regularization coefficients λ and γ as well as adjustable kernel parameters t and adjusted from the given set $\left\{10^{-4},10^{-3},10^{-2},\cdots ,10^{2},10^{3},10^{4}\right\}$. The dimension d of the subspace stack network was adjusted from the following set {5,10,15,⋯}.The local ratios $r_{b}$ and $r_{w}$ were empirically chosen as 0.9 for this study. The parameter description and setting of the HBD-SFREM are shown in Table 2. In this study, all experiments were repeated ten times and the statistical results are reported.

### 3.3. Evaluation Criteria

The proposal of a new algorithm needs to be evaluated using a series of criteria. This study selected five model evaluation metrics to comprehensively evaluate the HBD-SFREM. They are: model prediction accuracy rate (Acc), model prediction correct rate (Pre), model recall rate (Rec), and comprehensive evaluation metrics F-score and G-mean. All the above evaluation metrics were constructed by a confusion matrix. The confusion matrix is a table that visualizes the model predictions [39]. The PD speech diagnosis studied in this paper is a binary classification problem, thus the confusion matrix was constructed as shown in Table 3.

Based on the above definition of the confusion matrix, the evaluation metrics (EM) of the algorithmic model studied in this paper could be defined as:

- $Acc=\frac{TP+TN}{TP+FP+FN+TN}$;
- $Pre=\frac{TP}{TP+FP}$;
- $Rec=\frac{TP}{TP+FN}$;
- $Spe=\frac{TN}{FP+TN}$;
-
$G-mean=\sqrt{Rec*Spe}=\sqrt{\frac{TP}{TP+FN}*\frac{TN}{FP+TN}}\;$

- $F-score=2*\frac{Pre*Rec}{Pre+Rec}\;$;

### 3.4. Results and Analysis

In this part, the ablation method was used to verify the major innovation parts of the HBD-SFREM and then the representative feature extraction and feature selection algorithms were selected for comparison. Furthermore, existing feature reduction algorithms for PD speech recognition and two deep learning methods were also introduced in comparing with the proposed model. In the experiments, the hold-out method was used to divide the PD speech dataset: the dataset was randomly partitioned into three disjoint sets, including the training, validation, and test sets. As multiple speech segments (instances) were collected for each PD subject in the used dataset, instances from the same subject should be divided into the same set, to avoid the crossover of instances from the same subjects which could effectively respond to the authenticity of the results.

#### 3.4.1. Verification of the Effectiveness of HBD-SFREM

This section introduces the verification results of the innovation of HBD-SFREM, including the results of the BD-SFREM and those of the hierarchical space instance learning mechanism. It is worth noting that since the construction of the different hierarchy space is the basis for its learning mechanism, the validity of the hierarchical space instance learning mechanism could further prove the effectiveness of the construction of the different hierarchy instance space.

1. Verification of the BD-SFREM;

This part gives the results of both D-Spair and BD-SFREM. Two of the feature processing methods were chosen for constructing the D-Spair, and these are local discriminant preservation projection (LDPP) and Relief. To give a much clearer presentation of the results, some symbols should be defined below. Only-FE represents the mere usage of LDPP to process the features and Only-FS the Relief. D-Spair stands for the results of D-Spair module, while BD-SFREM represents the result of boosting dual-stage feature reduction pair ensemble module. (B) represents the affinity matrix of the binary mode in the feature extraction and (H) the heat kernel mode. The experiments constructed in this section were performed in the original instance space.

As shown in Table 4, for LSVT, Parkinson, and PSDMTSR, the BD-SFREM had the best results in Acc, Pre, Rec, G-mean, and F-score regardless of diverse classifier, while for SelfData, the BD-SFREM had the best results in Acc and Pre. In addition, the results of D-Spair and BD-SFREM were much more accurate than those of the Only-FS and Only-FE. Thus, the D-Spair module and BD-SFREM are effective. Three of the four datasets used in this paper are unbalanced datasets. From the experiment results in the above table, the BD-SFREM module is helpful in handling imbalanced instance datasets, especially for the LSVT, PSDMTSR, and Parkinson datasets, and the advantages of the BD-SFREM are more obvious. Since the quality of the self-collected dataset was lower than that of the public dataset, its model effectiveness was accordingly reduced. However, it can be improved by the IDEM mechanism, which is illustrated in next section.

2. Verification of the hierarchical space instance learning mechanism;

This section compares the results of the deep hierarchy instance space with those of the original instance space, and illustrates the effectiveness of the hierarchical space instance learning mechanism. (O) represents the results in the original instance space and (H) the results in the deep hierarchy instance space. Specifically speaking, Only-FS (O) stands for the results of the original instance space, and Only-FS (H) the results of the deep hierarchy instance space.

As shown in Table 5, the results of the deep hierarchy space instance (H) were improved for all PD speech datasets in diverse methods compared with the results of the original instance space (O). For LSVT, PSDMTSR, and SelfData, the results of (H) were obviously better than those of (O). For Parkinson, the results of (H) were also improved, though insignificantly. The last two columns of the table are the results of BD-SFREM, from which the results of (H) were obviously better than those of (O) in all datasets, with a maximum improvement rate of 9.53% on the LSVT dataset. Therefore, the hierarchical space instance learning mechanism in this paper is effective.

Table 6 shows the results of HBD-SFREM in different spaces (in which SVM (RFE) classifier is used). From the results in Table 6, we can see that the integrated output is always optimal, which further improves the generalization performance of the whole model.

#### 3.4.2. Comparison with the Representative Feature Processing Model

In this section, some representative feature processing methods, like mRMR, Pvalue, SVMRFE, PCA, and LDA, were selected to compare with the proposed model (HBD-SFREM. Because deep learning also acts as major feature processing methods, its two representative methods, namely deep belief network (DBN) and stacked encoder (SE), were compared with HBD-SFREM in this paper. To facilitate the results presentation, some symbols should be defined in the first place. HBD-SFREM (B) stands for the results in mode B, and HBD-SFREM (H) the results of mode H.

**Figure 3:**
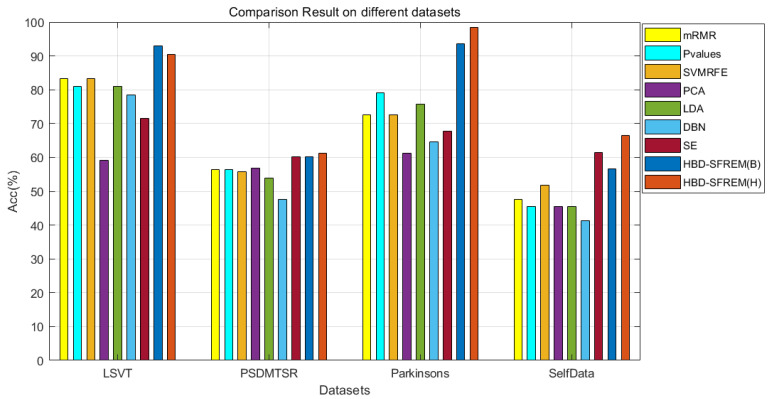
Comparison Results Using Different Datasets.

As shown in Table 7, the results of HBD-SFREM outperformed the algorithm reference groups on ACC and Pre, regardless of diverse datasets and classifiers. For the LSVT dataset, HBD-SFREM outperformed those reference groups on Rec, G-mean, and F-score. For the PSDMTSR and Parkinson datasets, the results of HBD-SFREM in G-mean and F-score were more accurate than those of reference groups. For SelfData, the results of the HBD-SFREM on Acc and Pre were better than its reference groups. To demonstrate the advantages of HBD-SFREM more clearly, the results of using SVM (RBF) classifier on different datasets are given in Figure 3, where the HBD-SFREM has achieved the best accuracy. In summary, HBD-SFREM outperformed the reference groups in most cases, which further verifies the effectiveness of HBD-SFREM.

**Figure 4:**
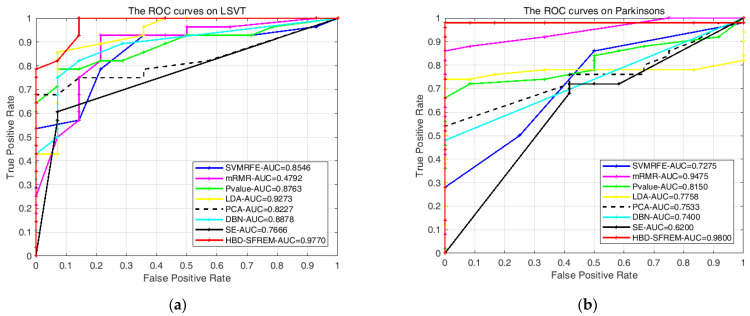

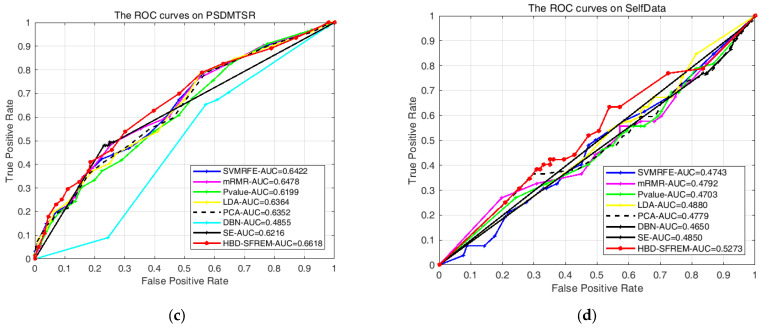
(**a**) Description of the ROC curves on LSVT; (**b**) description of the ROC curves on Parkinson; (**c**) description of the ROC curves on PSDMTSR; (**d**) description of the ROC curves on SelfData.

In addition, the ROC curves of all models on different datasets are shown in Figure 4. From Figure 4, we can see the area under curves (AUC) of HBD-SFREM is higher than the comparison models. It is worth noting that since SelfData is designed to simulate the real diagnosis environment of doctors, it is weaker in quality than the other three public datasets, but even under such conditions, the experimental result (AUC) shown in Figure 4 still proves that the HBD-SFREM is better than the comparative methods.

#### 3.4.3. Comparison with Relevant PD Speech Recognition Methods

HBD-SFREM primarily improves the accuracy of PD speech recognition. This section aims to show the effectiveness of the HBD-SFREM by comparing it with other PD speech FR algorithms. The algorithm reference groups are as follows:

(1) Relief-SVM [4]: Little used method in 2012, it involves first selecting four feature processing methods to process the features of the dataset, and then using Relief and SVM classifier with linear kernel function model (Relief-SVM) to learn to obtain a model.
(2) mRMR classifier [3]: This method was used by Sakar in 2018. In [3], feature selection is first performed using mRMR and then the prediction results voting or stacking strategies of seven classifiers are integrated.
(3) LDA-NN-GA [20]: This algorithm was proposed by L Ali and C Zhu in 2019. In [20]. The dataset is partitioned into a training set and a test set using the leave-one-out method (LOSO). Since each subject in the dataset contains multiple samples, the leave-one-out method here actually leaves all samples from one subject. Then, the feature dimension of the dataset is reduced using the LDA dimension reduction algorithm, and the BP neural network with genetic algorithm optimization is used to train the optimal prediction model (LDA-NN-GA).
(4) FC-SVM [6]: This algorithm was proposed by Cigdem O in 2018. In [6], the Fisher criterion (FC)-based feature selection method is used to rank feature weights, finally, the first K useful features are selected based on a threshold to input the classifier (SVM with RBF) for training to obtain the model.
(5) SFFS-RF [40]: This algorithm was proposed by Galaz Z in 2016. In this study, the sequential floating feature selection algorithm (SFFS) is adopted to process the data features, followed by inputting the processed results into the RF classifier to learn the prediction model.

Table 8 shows that HBD-SFREM always performed better than the other algorithms. For LSVT and Parkinson, the results were higher than those of the other algorithms, and the largest improvement rates in accuracy were 16.67% and 38.71%, respectively, demonstrating the advantages of HBD-SFREM. For SelfData and PSDMTSR, the results of HBD-SFREM were higher than the other algorithms in most cases, and the biggest improvement rates in accuracy were 22.37% and 22.27%, respectively. In addition, the experimental results of the comparison algorithms selected in this section were not as excellent as described in relevant studies, and the reason for this phenomenon is probably because the experimental conditions in this study were slightly different from those used in the reference group. For instance, the data diversity method differed from the method used by the authors in [20]. Additionally, the number of training data used in this study were less than that of [20]. In general, the larger the number of training data instances, the higher the prediction accuracy produced by the training model.

## 4. Discussion and Conclusions

HBD-SFREM has introduced an excellent dual-stage feature processing method that integrates the advantages of traditional feature extraction and feature selection algorithms. HBD-SFREM could generate high-quality features that are most useful to model learning, and thus achieve an early and accurate diagnosis of PD. These benefits can improve the identification accuracy as well as its stability. In addition, HBD-SFREM could be applied to small sample datasets of PD speech, including some unbalanced speech datasets. Experimental results demonstrate that the HBD-SFREM outperforms other existing algorithms of PD speech diagnosis.

Currently, publicly available PD speech datasets are relatively few. Three public PD speech datasets from UCI are introduced to validate the effectiveness as well as the innovativeness of the HBD-SFREM. In addition, this article also introduces the Chinese PD speech dataset collected by the authors. The experimental results indicate that HBD-SFREM achieves significantly better performance with the datasets studied. For all datasets, HBD-SFREM largely improves the diagnosis accuracy, especially on the Parkinson dataset. The degree of accuracy is enhanced by at least 19.36% compared to the other representative feature processing algorithms. At present, there are still relatively few fusion methods to study the selection and extraction of features for PD speech recognition, so this paper lays a good foundation for future research.

For future study, many more types of feature extraction and selection methods should be introduced into this research to develop and evaluate further effective algorithms. Besides, the improvement of the hierarchical space instance learning mechanism should be verified. As a framework algorithm, HBD-SFREM is different from other extraction and feature selection algorithms. Therefore, HBD-SFREM is rather valuable for reference and study in this field.

## Figures and Tables

**Table 1:** Basic information about datasets.

Database	Attributes					
	Patients	Healthy People	Instances	Features	Classes	Reference
LSVT	14	0	126	309	2	[36]
PSDMTSR	20	20	1040	26	2	[37]
Parkinson	24	8	195	23	2	[38]
SelfData	10	21	403	26	2	--

For the LSVT dataset, ’healthy people’ means the number of patients whose clinicians allowed ongoing rehabilitation, and ’patients’ mean the number of patients whose clinicians did not allow rehabilitation. For the SelfData dataset, the ‘healthy people’ denote the number of patients treated with the relevant medication and the ‘patients’ mean the number of patients treated with the relevant medication before.

**Table 2:** Parameter description and setting.

Parameter	Meaning	Parameter Setting
H	Layers of deep instance space	2
n	Numbers of independent instance space	3
λ	Penalty factor for $M^{T}(\gamma XAX^{T}-S_{DC})M$	10^−4^,10^−3^,…,10^4^
γ	Penalty factor for $M^{T}XAX^{T}M$	10^−4^,10^−3^,…,10^4^
t	Kernel parameter for affinity matrix	10^−4^,10^−3^,…,10^4^
k	Number of nearest neighbor instances in Z	5
d	Dimension after FR	5,10,15,…
P	Instance output rate of each hierarchical	0.8

**Table 3:** Confusion matrix for PD speech recognition problem.

		Prediction Labels	
		Positive (P)	Negative (N)
Real label	Positive (P)	TP	FN
	Negative (N)	FP	TN

**Table 4:** Results of the validation of the algorithm using the ablation method (%).

		Methods	Only-FS	Only-FE (B)	Only-FE (H)	D-Spair (B)	D-Spair (H)	BD-SFREM (B)	BD-SFREM (H)
Datasets/ EM/Classifier									
LSVT	ACC	SVM (linear)	78.57	78.57	78.57	83.33	83.33	85.71	92.86
		SVM (RBF)	76.19	73.81	71.43	83.33	85.71	83.33	90.48
	pre	SVM (linear)	95.24	82.76	91.30	88.89	88.89	96.00	100.00
		SVM (RBF)	95.00	90.48	78.57	92.00	92.31	96.00	96.15
	Rec	SVM (linear)	71.43	85.71	75.00	85.71	85.71	85.71	89.29
		SVM (RBF)	67.86	67.86	78.57	82.14	85.71	85.71	89.29
	G-mean	SVM (linear)	81.44	74.23	80.18	82.07	82.07	89.21	94.49
		SVM (RBF)	79.38	76.26	67.01	83.91	85.71	89.21	91.05
	F-score	SVM (linear)	81.63	84.21	82.35	87.27	87.27	90.57	94.34
		SVM (RBF)	79.17	77.55	78.57	86.79	88.89	90.57	92.59
PSDMTSR	Acc	SVM (linear)	45.19	54.81	52.56	55.77	56.41	58.07	58.33
		SVM(RBF)	46.79	55.77	55.77	55.77	56.73	57.37	58.97
	Pre	SVM (linear)	42.11	57.89	54.88	60.98	60.42	65.43	61.61
		SVM (RBF)	46.21	59.18	59.78	5918	61.29	60.18	60.45
	Rec	SVM (linear)	45.19	35.26	28.85	32.05	37.18	33.97	44.23
		SVM (RBF)	47.44	37.18	35.26	37.18	36.54	43.59	51.92
	G-mean	SVM (linear)	40.74	51.20	46.19	50.47	53.03	52.80	56.60
		SVM (RBF)	46.16	52.58	51.86	52.58	53.02	55.69	58.55
	F-Score	SVM (linear)	31.87	43.82	37.82	42.02	46.03	44.73	51.49
		SVM (RBF)	42.36	45.67	44.35	45.67	45.78	50.56	55.86
Parkinson	Acc	SVM (linear)	59.68	66.13	66.13	67.74	79.03	96.77	95.16
		SVM (RBF)	61.29	59.68	61.29	67.74	62.90	83.87	79.03
	Pre	SVM (linear)	90.32	100.00	100.00	100.00	100.00	100.00	100.00
		SVM (RBF)	84.21	90.32	84.21	82.61	80.00	97.62	93.02
	Rec	SVM (linear)	56.00	58.00	58.00	60.00	74.00	96.00	94.00
		SVM (RBF)	64.00	56.00	64.00	76.00	72.00	82.00	80.00
	G-mean	SVM (linear)	64.81	76.16	76.16	77.46	86.02	97.98	96.95
		SVM (RBF)	56.57	64.81	56.57	50.33	42.43	86.70	77.46
	F-score	SVM (linear)	69.14	73.42	73.42	75.00	85.06	97.96	96.91
		SVM (RBF)	72.73	69.14	72.73	79.17	75.79	89.13	86.02
Self Data	Acc	SVM (linear)	47.55	44.76	45.45	58.04	55.24	58.74	58.74
		SVM (RBF)	45.45	43.36	45.45	46.85	46.15	49.65	58.04
	Pre	SVM (linear)	35.06	33.33	34.15	38.89	36.36	40.54	40.00
		SVM (RBF)	33.75	32.53	34.52	35.00	34.57	34.38	33.33
	Rec	SVM (linear)	51.92	51.92	53.85	26.92	30.77	28.85	26.29
		SVM (RBF)	51.92	51.92	55.77	53.85	53.85	42.31	15.38
	G-mean	SVM (linear)	48.37	45.95	46.79	45.18	46.15	46.77	45.50
		SVM (RBF)	46.56	44.69	46.97	48.04	50.39	47.73	35.60
	F-score	SVM (linear)	41.86	40.60	41.79	31.82	33.33	33.71	32.18
		SVM (RBF)	40.91	40.00	42.65	42.42	42.11	37.93	24.05

**Table 5:** Verification of hierarchy space instance learning mechanism (%).

		Methods	Only-FS		Only-FE (B)		D-Spair (B)		BD-SFREM (B)	
Datasets/ EM/Classifier			(O)	(H)	(O)	(H)	(O)	(H)	(O)	(H)
LSVT	ACC	SVM (linear)	78.57	80.95	78.57	85.71	83.33	85.71	85.71	85.71
		SVM (RBF)	76.19	83.33	73.81	83.33	83.33	85.71	83.33	92.86
	pre	SVM (linear)	95.24	95.45	82.76	95.83	88.89	95.83	96.00	100.00
		SVM (RBF)	95.00	92.00	90.48	88.89	92.00	92.31	96.00	96.30
	Rec	SVM (linear)	71.43	75.00	85.71	82.14	85.71	82.14	85.71	85.71
		SVM (RBF)	67.86	82.14	67.86	85.71	82.14	85.71	85.71	92.86
	G-mean	SVM (linear)	81.44	83.45	74.23	87.34	82.07	87.34	88.10	85.71
		SVM (RBF)	79.38	83.91	76.26	82.07	83.91	85.71	88.10	92.86
	F-score	SVM (linear)	81.63	84.00	84.21	88.46	87.27	88.46	89.21	88.89
		SVM (RBF)	79.17	86.79	77.55	87.27	86.79	88.89	89.21	94.55
PSDMTSR	Acc	SVM (linear)	45.19	48.08	54.81	58.01	55.77	57.69	58.01	57.05
		SVM(RBF)	47.44	52.88	55.77	57.37	55.77	57.37	57.37	60.26
	Pre	SVM (linear)	42.11	47.86	57.89	62.89	60.98	65.79	65.43	60.19
		SVM(RBF)	64.22	56.92	59.18	60.36	59.18	60.36	60.18	61.11
	Rec	SVM (linear)	25.64	42.95	35.26	39.10	32.05	32.05	33.97	41.67
		SVM(RBF)	44.87	23.72	37.18	42.95	37.18	42.95	43.59	56.41
	G-mean	SVM (linear)	40.74	47.80	51.20	54.84	50.47	51.68	52.80	54.94
		SVM(RBF)	58.01	44.11	52.58	55.53	52.58	55.53	55.69	60.13
	F-Score	SVM (linear)	31.87	45.27	43.82	48.22	42.02	43.10	44.73	49.24
		SVM(RBF)	52.83	33.48	45.67	50.19	45.67	50.19	50.56	58.67
Parkinson	Acc	SVM (linear)	59.68	72.58	66.13	74.19	67.74	82.26	96.77	85.48
		SVM (RBF)	61.29	67.74	59.68	70.97	67.74	67.74	83.87	85.48
	Pre	SVM (linear)	90.32	86.67	100.00	100.00	100.00	100.00	100.00	100.00
		SVM (RBF)	84.21	85.71	90.32	79.63	82.61	82.61	97.62	100.00
	Rec	SVM (linear)	56.00	78.00	58.00	68.00	60.00	78.00	96.00	82.00
		SVM (RBF)	64.00	72.00	56.00	86.00	76.00	76.00	82.00	82.00
	G-mean	SVM (linear)	64.81	62.45	76.16	82.46	77.46	88.32	97.98	90.55
		SVM (RBF)	56.57	60.00	64.81	26.77	50.33	50.33	86.70	90.55
	F-score	SVM (linear)	69.14	82.11	73.42	80.95	75.00	87.64	97.96	90.11
		SVM (RBF)	72.73	78.26	69.14	82.69	79.17	79.17	89.13	90.11
Self Data	Acc	SVM (linear)	47.55	48.25	44.76	45.45	58.04	47.55	58.74	62.94
		SVM(RBF)	45.45	46.15	43.36	45.45	46.85	50.35	49.65	49.65
	Pre	SVM (linear)	35.06	35.53	33.33	33.75	38.89	35.05	40.54	47.06
		SVM (RBF)	33.75	33.77	32.53	34.15	35.00	35.82	34.38	32.14
	Rec	SVM (linear)	51.92	51.92	51.92	51.92	26.92	51.92	28.85	15.38
		SVM (RBF)	51.92	50.00	51.92	53.85	53.85	46.15	42.31	34.62
	G-mean	SVM (linear)	48.37	48.95	45.95	46.56	45.18	48.36	46.77	37.23
		SVM (RBF)	46.56	46.88	44.69	46.79	48.04	49.34	47.73	44.90
	F-score	SVM (linear)	41.86	42.19	40.60	40.91	31.82	41.86	33.71	23.19
		SVM (RBF)	40.91	40.31	40.00	41.79	42.42	40.34	37.93	33.33

**Table 6:** Verification of the integration output (%).

	Hierarchical Space	Original Space	Deep Space 1	Deep Space 2	$P_{Final}$
Datasets/EM					
LSVT	ACC	83.33	92.86	85.71	92.86
	pre	92.00	96.30	95.83	96.30
	Rec	82.14	92.86	82.14	92.86
	G-mean	83.91	92.86	87.34	92.86
	F-score	86.79	94.55	88.46	94.55
PSDMTSR	ACC	57.37	54.49	60.26	60.26
	pre	60.18	56.36	61.11	61.11
	Rec	43.59	39.74	56.41	56.41
	G-mean	55.69	52.45	60.13	60.13
	F-score	50.56	46.62	58.67	58.67
Parkinson	ACC	83.87	82.26	85.48	93.55
	pre	97.62	67.90	1.00	97.62
	Rec	82.00	92.00	82.00	94.00
	G-mean	86.70	61.91	90.55	92.83
	F-score	89.13	89.32	90.11	95.92
SelfData	ACC	49.65	49.65	56.64	56.64
	pre	34.38	32.14	40.74	40.74
	Rec	42.31	34.62	42.31	42.31
	G-mean	47.73	44.90	52.37	52.37
	F-score	37.93	33.33	41.51	41.51

**Table 7:** Comparison with representative feature processing algorithms (%).

		Methods	mRMR	Pvalue	SVMRFE	PCA	LDA	DBN	SE	HBD-SFREM	
Datasets/EM/Classifier										(B)	(H)
LSVT	ACC	SVM (linear)	76.19	83.33	73.81	83.33	78.57	78.57	71.43	88.10	92.86
		SVM (RBF)	83.33	80.95	83.33	69.05	80.95			92.86	90.48
	pre	SVM (linear)	100.00	100.00	94.74	95.65	91.30	95.24	94.44	100.00	100.00
		SVM (RBF)	100.00	91.67	83.87	100.00	95.45			96.30	96.15
	Rec	SVM (linear)	64.29	75.00	64.29	78.57	75.00	71.43	60.71	89.29	89.29
		SVM (RBF)	75.00	78.57	92.86	53.57	75.00			92.86	89.29
	G-mean	SVM (linear)	80.18	86.60	77.26	85.42	80.18	81.44	75.08	94.48	94.49
		SVM (RBF)	86.60	82.07	77.26	73.19	83.45			92.86	91.05
	F-score	SVM (linear)	78.26	85.71	76.60	86.27	82.35	81.63	73.91	94.34	94.34
		SVM (RBF)	85.71	84.62	88.14	69.77	84.00			94.55	92.59
PSDMTSR	Acc	SVM (linear)	48.08	46.47	52.56	57.05	48.40	47.60	60.26	61.22	66.35
		SVM(RBF)	56.41	56.41	55.77	56.73	53.85			60.26	61.22
	Pre	SVM (linear)	47.86	45.99	56.25	63.10	47.13	46.27	64.29	69.23	72.57
		SVM(RBF)	62.82	59.09	57.38	58.27	54.76			61.11	64.00
	Rec	SVM (linear)	42.95	.40.38	23.08	33.97	26.28	29.81	64.15	40.38	52.56
		SVM(RBF)	31.41	41.67	44.87	47.44	44.23			56.41	51.28
	G-mean	SVM (linear)	47.80	46.07	43.51	52.18	43.05	44.15	58.58	57.56	64.90
		SVM(RBF)	50.57	54.45	54.69	55.96	52.98			60.13	60.41
	F-Score	SVM (linear)	45.27	43.00	32.73	44.17	33.74	36.26	53.73	51.01	60.97
		SVM(RBF)	41.88	48.87	50.36	52.30	48.94			58.67	56.94
Parkinson	Acc	SVM (linear)	72.58	82.26	80.65	64.52	69.35	64.52	67.74	96.77	95.16
		SVM (RBF)	72.58	79.03	72.58	61.29	75.81			93.55	98.39
	Pre	SVM (linear)	100.00	100.00	80.65	100.00	96.97	100.00	87.50	100.00	100.00
		SVM (RBF)	100.00	93.02	78.95	76.00	100.00			97.92	100.00
	Rec	SVM (linear)	74.00	78.00	100.00	56.00	64.00	56.00	70.00	96.00	94.00
		SVM (RBF)	66.00	80.00	90.00	76.00	70.00			94.00	98.00
	G-mean	SVM (linear)	86.02	88.32	00.00	74.83	76.59	74.83	63.90	97.98	96.95
		SVM (RBF)	81.24	77.46	00.00	00.00	83.67			92.83	98.99
	F-score	SVM (linear)	85.06	87.64	89.29	71.79	77.11	71.79	77.78	97.96	96.91
		SVM (RBF)	79.52	86.02	84.11	76.00	82.35			95.92	98.99
Self Data	Acc	SVM (linear)	48.25	44.76	60.14	48.25	45.45	41.26	61.54	64.34	61.54
		SVM(RBF)	47.55	45.45	51.75	45.45	45.45			56.64	66.43
	Pre	SVM (linear)	35.90	34.12	36.84	35.90	35.87	34.00	42.86	53.85	42.86
		SVM (RBF)	35.80	34.52	33.33	34.88	35.87			40.74	70.00
	Rec	SVM (linear)	53.85	55.77	13.46	53.85	63.46	65.38	17.31	13.46	17.31
		SVM (RBF)	55.77	55.77	32.69	57.69	63.46			42.31	13.46
	G-mean	SVM (linear)	48.25	44.76	60.14	48.25	45.45	42.38	38.76	35.46	38.76
		SVM (RBF)	49.25	46.31	34.18	49.25	47.24			52.37	36.08
	F-score	SVM (linear)	45.32	44.69	41.83	45.04	46.43	44.74	24.66	21.54	24.66
		SVM (RBF)	43.08	42.34	19.72	43.08	45.83			41.51	22.58

**Table 8:** Comparison of PD speech dataset processing algorithms (%).

	Datasets	LSVT	PSDMTSR	Parkinson	SelfData
Methods					
HBD-SFREM (B)	SVM (linear)	92.86	61.22	96.77	64.34
	SVM (RBF)	92.86	60.26	93.55	56.64
HBD-SFREM (H)	SVM (linear)	92.86	66.35	95.16	61.54
	SVM (RBF)	90.48	61.22	98.39	66.43
Relief [4]	SVM (linear)	78.57	45.19	59.68	47.55
	SVM (RBF)	76.19	47.44	61.29	45.45
mRMR [3]	SVM (linear)	76.19	48.08	72.58	48.25
	SVM (RBF)	83.33	56.41	72.58	47.55
LDA-NN-GA [20]		81.42	61.38	80.83	63.00
ReliefF-FC-SVM (RBF) [6]		82.54	61.38	81.67	62.67
SFFS-RF [40]		81.64	60.63	80.83	60.00

## Data Availability

Three publicly available datasets are used in this paper and they can be downloaded from: www.archive.ics.uci.edu/ml/index.php (accessed on 24 November 2021). The code used in this study can be found at: https://github.com/YangMingYaoo/Deep-Embedded-hybrid-FR-algorithm-about-parkinsons-disease.git, (accessed on 24 November 2021).
